# Refinements in the use of silicone oil as an intraocular tamponade

**DOI:** 10.1038/s41433-024-03027-y

**Published:** 2024-03-28

**Authors:** D. Ian Wilson, Andrew D. te Water Naude, Martin P. Snead

**Affiliations:** 1 Department of Chemical Engineering and Biotechnology, University of Cambridge, Philippa Fawcett Drive, Cambridge, CB3 0AS UK; 2 Vitreoretinal Service, University of Cambridge, Addenbrookes Hospital, Hills Road, Cambridge, CB2 2QQ UK

**Keywords:** Conferences and meetings, Education

## Abstract

It is over 60 years since Paul Cibis et al. reported the experimental use of liquid silicone in the surgical management of retinal detachment. Initial experiences were complicated by significant side-effects associated with the impurities in the non-medical grade commercial silicone oils deployed at the time. These were substantially reduced (but not eliminated) by the adoption of refined high-viscosity medical grade silicone oils. Two of the major complications associated with silicone tamponade are (i) the variability of focus due to its movement and higher refractive index, and (ii) progressive emulsification, particularly with low viscosity oils. This article reviews recent and ongoing research on the causes of emulsification of intra-ocular silicone oil to understand the causes better and thereby reduce this risk, especially for those eyes where permanent tamponade is the only current option for retaining vision.

## Abstract (zh)

自Paul Cibis等人实验性地将液态硅油用于视网膜脱落的手术治疗以来, 已过去 60多年。由于当时使用的非医用级商业硅油中含有杂质, 会产生严重副作用, 因此最初的经验难以确定。采用精制的高粘度医用级硅油后, 副作用大大减少 (但并未完全消除) 。与硅油填塞相关的两个主要并发症是: (i) 由于硅油的移动和较高折射率而导致眼部焦距变化；(ii) 硅油逐渐乳化, 尤其是低粘度硅油。本文回顾了近期和正在进行的关于眼内硅油乳化原因的研究, 以更好地了解其原因, 从而降低该风险, 尤其是对于那些病人来说永久性填塞是目前唯一保留视力的方法。

## Introduction

It is over 60 years since Paul Cibis, Ed Okun and colleagues [1] reported the experimental use of liquid silicone in the surgical management of retinal detachment. Initial experiences were complicated by significant side-effects associated with the impurities in the non-medical grade commercial silicone oils deployed at the time and these were subsequently substantially reduced (but not eliminated) by the adoption of refined high-viscosity medical grade silicone. Two of the major complications associated with silicone tamponade are the variability of focus due to its movement and higher refractive index and progressive emulsification, particularly with low viscosity oils. For these reasons, removal of silicone oil tamponade is generally advisable as soon as the retina has been successfully stabilised. In a small minority of patients (particularly those with complex recurrent detachment), permanent silicone tamponade sometimes offers the only chance of salvage and stabilisation of vision and in such instances deployment of high purity, high viscosity oil is recommended [2] but progressive emulsification persists in some patients. Furthermore, the recent trend towards small gauge instrumentation has led some centres to revert back to use of low viscosity oils for ease of delivery and removal through narrow gauge cannulas but offset by risk of earlier and more extensive emulsification. This article reviews recent and ongoing research on the causes of emulsification of intra-ocular silicone oil to better understand and reduce this risk, especially for those eyes where permanent tamponade is the only current option for retaining vision.

## Recent developments

**Fig. 1:**
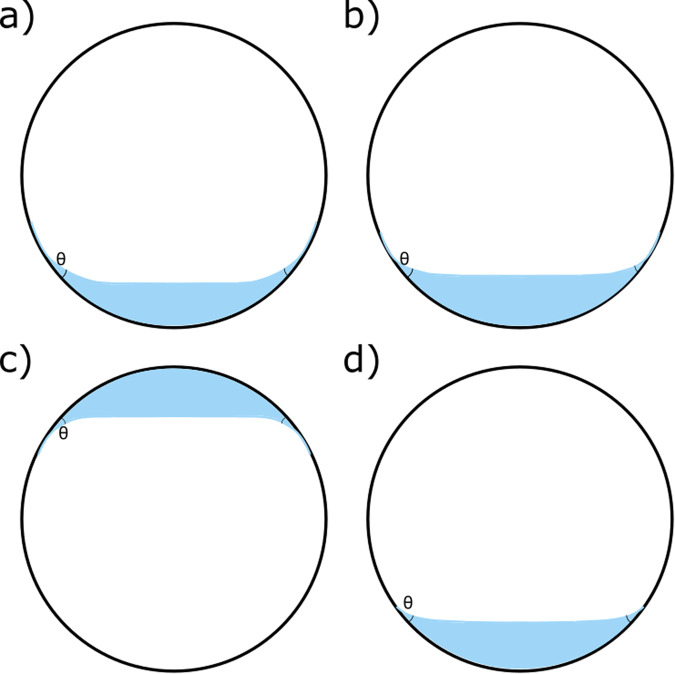
Shape of tamponade-aqueous interface shape in a 24 mm sphere, representative of the human eye, for 90% level of fill. **a** SO, density 0.965 g cm^−3^, contact angle, = 20°, interfacial tension (IFT) 35.5 mN m^−1^, 10.2 mm; **b** SO in (**a**) with = 15°; **c** heavy SO, density 1.06 g cm^−3^, = 20°, IFT 40.8 mN m^−1^, 8.2 mm; **d** gaseous tamponade (SF_6_), density 0.0065 g cm^−3^, = 20°, IFT 72.8 mN m^−1^, 2.7 mm. Aqueous phase in blue. Physical properties taken from the literature, values and methodology reported in Supplementary Material.

Intraocular tamponades (IOTs) are used in vitreoretinal surgery to tamponade and maintain retinal break closure until the retinopexy has matured to full strength. Gaseous intraocular tamponades undergo spontaneous resolution by absorption into the blood stream (their volume being taken up by aqueous phase) whereas silicone tamponades require a second surgical intervention for their removal from the eye. Gaseous intraocular tamponades in common use include air, sulphur hexafluoride (SF_6_), perfluoroethane (C_2_F_6_) and perfluorooctane (C_3_F_8_). Liquid tamponades (often silicone oil (SO): polydimethyl siloxane, PDMS, with chain lengths of order 37–65 kDa [3]; corresponding to 450–850 monomers) are effectively immiscible with water. The eye continues to generate the aqueous phase from the ciliary body epithelium and aqueous leaves the eye principally via anterior chamber drainage system but also posteriorly (uveo-scleral outflow). In the vitreous cavity the aqueous phase forms a curved interface between either the gas or silicone tamponade. The shape of the static interface is determined by the level of fill, the contact angle between the two phases, *θ*, and the retinal wall, the local surface topology (e.g. smooth – lens; undulating – retina; shaped – scleral buckle) and the liquid properties: notably the difference in density of the two fluids, ∆*ρ*, and the interfacial tension, *γ*. Figure 1 illustrates the impact of these properties on interface shape for tamponade fluids in regular use and a 90% level of fill. The characteristic length scale is the capillary length, ${l}_{{{{{{\rm{c}}}}}}}\equiv \sqrt{\gamma /g\Delta \rho }$, where *g* is the gravitational acceleration, and the position of the tamponade is determined by its density relative to aqueous.

Developments in IOT technology over the last decade include the development of new tamponade materials, particularly silicone oil-based mixtures which are denser than water (e.g. Densiron 68, [4]) and hydrogel-based intravitreal tamponades [5, 6] with the objective of effectively tamponading inferior retinal breaks (on the lower part of the eyeball) as an alternative to the combination of PDMS IOTs and a scleral buckle. More recently, foldable capsular vitreous bodies (FCVBs) [7, 8] have been described to function as an intraocular balloon, inserted after pars planar vitrectomy then filled with liquid, providing a sterile and immobile tamponade.

Other studies include investigations of tamponade biocompatibility [9] and interactions between tamponade liquids and the aqueous phase.

## Silicone oil emulsification (SOE)

The observation of emulsification continues to attract attention, as the origins of this phenomenon are still not well understood. In SOE, small droplets of SO are generated in the aqueous phase, impairing vision and potentially leading to complications. Table 1 summarises retrospective studies of tamponade complications over the past decade. Most of the studies featured small sample sizes or were drawn from one hospital or patients of one surgeon. The probability of emulsification in large studies is approximately 1 in 20 (5%). The question posed at the COS 2023 conference ‘Engineering and the Eye’ was ‘Why is the rate so low?’ and this paper focuses on the advances in understanding of the origins of SOE.

**Table 1:** Summary of recent clinical studies of SOE.

Number of patients	Emulsification detected (%)	Silicone oil viscosity (cSt)	Inspection method	Emulsification time (months)			Source
				Median	Mean	S.D.	
379	8.44	1000	Patient record study	12.4	13.1	4.8	[31]
2619	5.47	1000	Patient record study	10.7	15.8	15.5	[32]
184	4.99	5000	Patient record study	21.2	25.9	15.5	[32]
24	37.5	1000	SD-OCT	3	4	2	[13]
50	63.6	1300	Slit lamp, gonioscopy, indirect ophthalmoscopy	6	6	3.5	[14]
50	40	5700	Slit lamp, gonioscopy, indirect ophthalmoscopy	6	4.6	2.9	[14]
4	50/100^b^	1300	Electrostatic counting^a^	15	18.7	5.1	[28]
5	80/100^b^	5500	Electrostatic counting^a^	12	10.2	7.5	[28]
38	100	5700	Electrostatic counting^a^	—	6.3	3.2	[11]
118	100^c^	5700	Ultrasound biomicroscopy	—	5.5	3.3	[12]

^a^Electrostatic counting (or electrozone method; Coulter counting) on the first 2 ml of aqueous washout from the eye following silicone oil removal. Changes in conductivity of the fluid as it flows between two electrodes correlates to the size of a silicone oil droplet present. The time courses presented for these studies are the time period of SO in the eye.

^b^Chan et al. (ref. [28]) reported a 100% emulsification rate when the electrostatic counting method was used. The first detection fraction reported is for emulsification being visible in a clinical setting before silicone oil removal

^c^Silicone oil was not removed from the eye until emulsification was detected by ultrasound biomicroscopy.

Table 1 shows that the average time for SOE to be observed in large studies increases with time for more viscous oils. The difference is not statistically significant, however. Chan et al. [10] reported the converse for their small (*N* = 9) study, which focused on the of use of a Coulter Counter (electrozone method) to determine the presence and size of SO droplets, while Yu et al. [11] reported noticeably shorter times (of order 6 months) for detection of SOE. These durations were similar to those reported by ultrasound biomicroscopy [12], SD-OCT [13] and slit lamp, gonioscopy and indirect ophthalmoscopy [14]. The method of inspection affects the detection efficiency noticeably. Emulsification is normally identified and quantified by optical observation, which sets a lower limit on the droplet size that can be detected, of approximately 30 μm. Chan et al. [10] and Yu et al. [11] measured droplet sizes in extracted fluids using techniques including laser light scattering and electrozone sensing, and reported sizes between 1 and 12 μm (with many 1–2 μm droplets), suggesting that SOE could be more prevalent than reported as smaller droplets are not detected by visual inspection. Romano et al. [15] recently proposed a grading system for SOE to allow systematic comparison between different studies.

Recent years have seen an increasing trend towards small gauge vitrectomy instrumentation, needing greater force for transfer of viscous oils and resulting in slower delivery: the scope for avoiding SOE by using highly viscous oils is limited. This has led to some centres reverting back to the use of low viscosity silicone oils and a resurgence of the attendant problems of rapid emulsification. Understanding the reasons for emulsification, thereby allowing mitigation to be planned for likely cases, has driven much of the research in this area.

The mechanism(s) causing SOE have yet to be established. Silicone oil and other immiscible liquids will form droplets in the aqueous phase when the interface between the two fluids becomes unstable. Adsorption of proteins and other surface active species at the interface will reduce the interfacial tension (IFT) – a measure of the work required to deform the interface - and promote interface breakup. Figure 1 shows how reducing the IFT changes the shape of the stable interface.

**Fig. 2:**
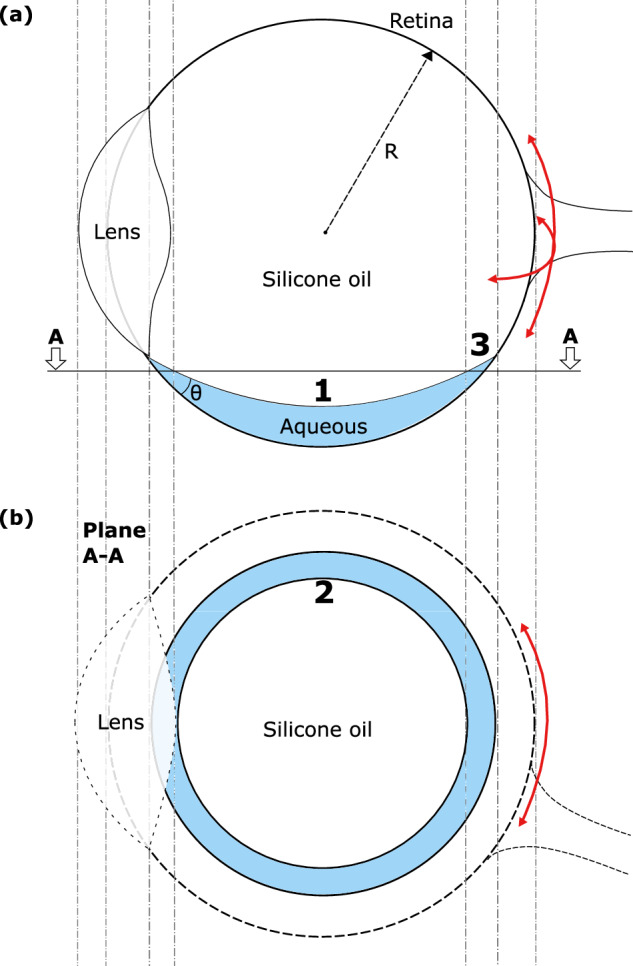
Schematic of SO tamponade (less dense than aqueous) showing regions where different emulsification mechanisms: (1) bulk instability; (2) thin film instability; (3) contact line motion, are expected to occur. **a** Vertical section through midplane; **b** horizontal section through plane AA.

The flow conditions in the eye which promote breakup are not well understood. Motion of the two fluids in the posterior segment (see Fig. 2) is driven by wall drag, where the saccadic motion of the eyeball accelerates the fluid in contact with the retina. The local change in velocity is determined by the dynamic viscosity of the fluid (the ratio of viscosity to density, *ν*) which differs by a factor of ~10^3^ between the aqueous and IOT phases. This leads to interfacial deformation. Detailed modelling of these flows, including computational fluid dynamics (CFD) simulations of the eyeball geometry, have been reported [16].

**Fig. 3:**
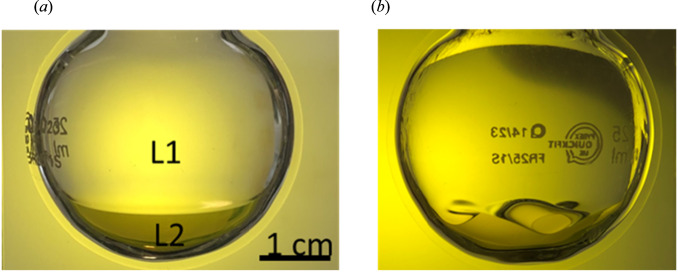
Photographs showing SO-aqueous interface in a 20 mm radius round bottomed flask. **a** Stationary and **b** strongly agitated, saccadic motion amplitude 36°, 600° s^−1^. Experimental conditions: 1 Pa s silicone oil (L1) and 1 wt.% TX-100 saline solution (L2), volume ratio 91:9 SO: aqueous. [23], reproduced with permission.

**Fig. 4:**
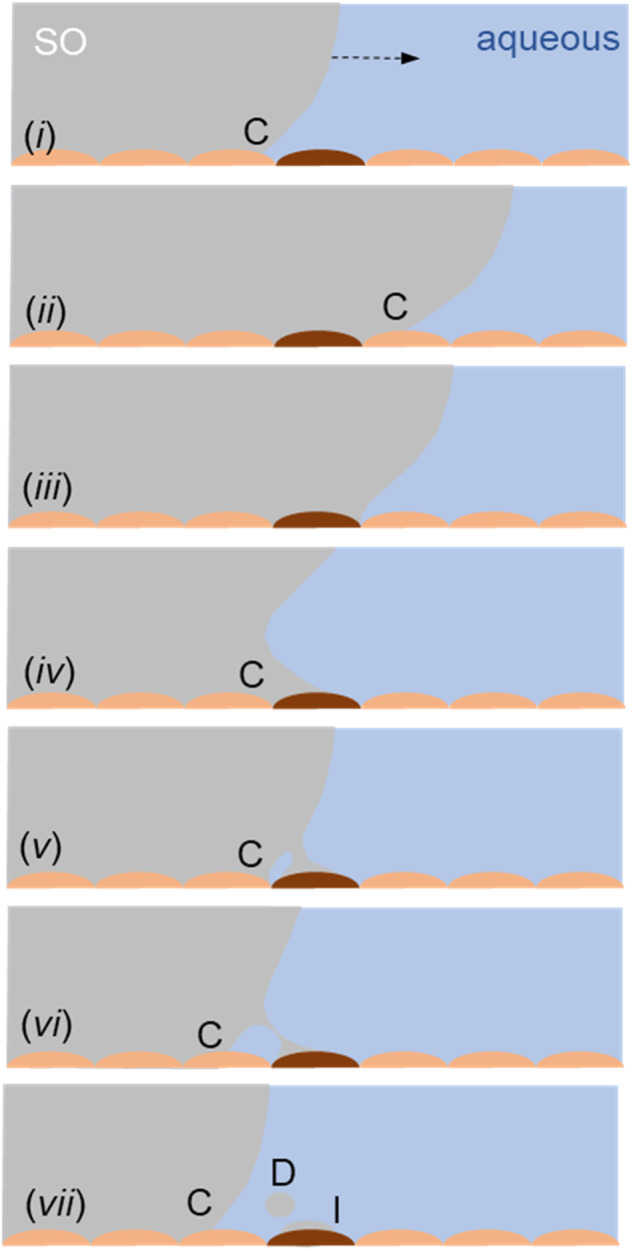
Cartoon showing formation of a droplet by pinning of the SO-aqueous contact line (C) on the retinal surface at a preferentially wetting site (in brown). The contact line moves across the site in (i–iii). As it retreats (iv-vi) it forms a filament which in this case (vii) ruptures to form a droplet (D) and an isolated SO drop (I). Alternately, a large isolated drop could be formed which subsequently gives daughter droplets as a result of high shear rates in the aqueous film.

Figure 2 shows the three regions where different interfacial breakup mechanisms could be expected to occur:

1. Bulk deformation. Here, saccadic motion generates an unstable interface (Fig. 3) which is thought to shed droplets and is often cited as the cause of SOE [17]. Wang et al. [18, 19] have shown experimentally that the interface deformation induced by saccadic motion under simulated physiological conditions (saccade speeds, presence of surfactants, surface features such as the lens and scleral buckle) is not strong enough to cause emulsification of standard surgical SOs. This finding is consistent with the relatively low occurrence of SOE: if this mechanism was responsible, the expected probability would be closer to 100%.
2. Thin film rupture. Here, the aqueous layer at the side of the eye is subject to high shear rates as it transfers momentum to the SO sac. Chen et al. [20] postulated that the stability of the interface, when reduced by adsorption of surface active species, would lead to emulsification. The criterion for the stability of a thin layer of a less viscous liquid between a wall subject to periodic motion and a more viscous fluid was investigated by Isakova et al. [21], but these workers did not consider the ratio of viscosities (of order 10^3^) which arise with IOTs associated with SOE. Moreover, these films are expected to present with all liquid tamponades.
3. Contact line disturbances. Wang et al. [19] observed droplet formation occasionally, in the region near the moving contact line. They hypothesised that emulsification could arise from motion of the two-liquid contact line across heterogeneous surface features (see Fig. 4). The motion of a two fluid contact line across surface heterogeneities, either arising from surface morphology or wettability, is the subject of active research in the fluid mechanics and microfluidics communities [22]. Wang [23] postulated that variations in the retinal surface, either arising from local morphology (e.g. scarring) or chemistry (adsorption of contaminants from the tamponade; local physiology) which rendered the surface more strongly wetting towards SO, could result in pinning of the contact line at these features, resulting in isolation of individual droplets, and their subsequent detachment when exposed to the high shear rates generated in the aqueous film.

**Fig. 5:**
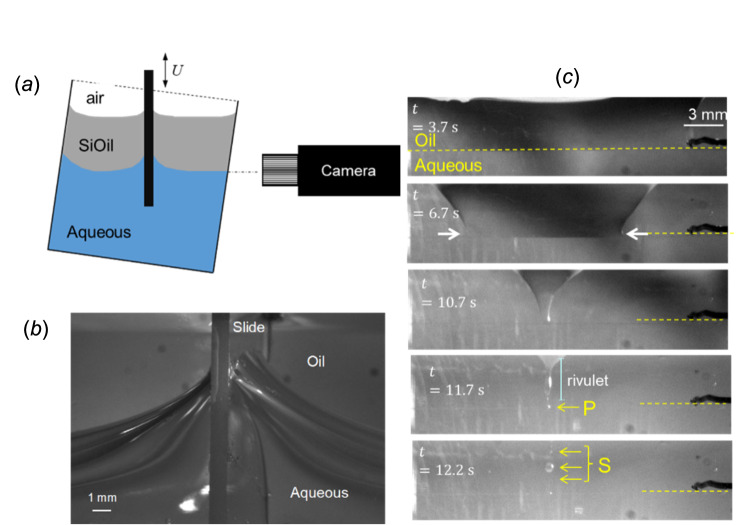
Experimental investigation of a SO-aqueous contact line moving across a line of SO-wetting dots. **a** Experimental configuration: more information is given in the Supplementary Information. A glass microscope slide is initially plunged downwards at speed *U* so that the contact line is below its equilibrium location shown in (**b**). The contact line then climbs back up, the aqueous displacing the SO, recorded in the photographs in (**c**). The times in (**c**) indicate the time elapsed since the slide was moved downwards. The horizontal dashed yellow line indicates the location of a line of 50 μm diameter silane (trichloro-1H,1H,2H,2H-perfluoro-octyl-silane) dots on the slide. At *t* = 3.7 s the contact line has reached the line of dots as it moves upwards. It is pinned at the line (*t* = 6.7 s) and then detaches from the final dot, forming a rivulet (*t* = 11.7 s) which can break to give mobile drops (marked S) and stranded drops (*e.g*., P) which could then be sheared off subsequently (see Fig. 2). The shear rates required to cause detachment are discussed in Supplementary Material.

Figure 5 shows some results of a synthetic experimental case, where motion of the contact line between the SO and aqueous phases across a solid surface featuring small hydrophobic patches results in droplet formation at the patches. Droplet formation was found to be determined by contact line speed, patch chemistry, patch shape and orientation (see Supplementary Information): these factors offer an explanation for the variation in observation of SOE between different individuals. It also offers insight into why the extensional viscosity of the SO can play a role in emulsification, as reported by Williams et al. [24]: the stretching of a droplet in a shear flow until it forms a neck which then breaks (Fig. 4(vi)) is governed by the shear stress imposed by the aqueous liquid and the extensional viscosity of the tamponade fluid. Wang did not observe droplet detachment often in these tests: this is consistent with the findings in studies of the detachment of silicone oil droplets from partially wetting (glass) surfaces by a shear flow of water and reported threshold relative velocities for droplet detachment [25], which Wang’s studies did not reach (see Supplementary Information). Testing this hypothesis for the parameter range of interest is the subject of ongoing research in our group.

Mechanisms such as (3), which feature interactions at the small scale, mean that they can be probed using simplified geometries and the full complexity of the flow patterns generated in the eye do not need to be replicated, whereas studies of bulk mechanisms (e.g. (1)) do need to replicate local shear rates. The flow patterns generated by the saccadic motion in the eye are complex [26, 27] and some researchers have employed simplified 1-D and 2-D geometries to study SOE. Translating the results from these model systems to clinical practice is challenging as the flow patterns often do not map directly to the quasi-spherical geometry of the eye. Chan et al. [28] employed a horizontal cylinder (diameter 20 mm, length 20 mm) to study the effect of factors such as a scleral buckle on emulsification, but did not consider how the circulation flow and boundary layers generated by the moving walls are very different from those in the eye.

**Fig. 6:**
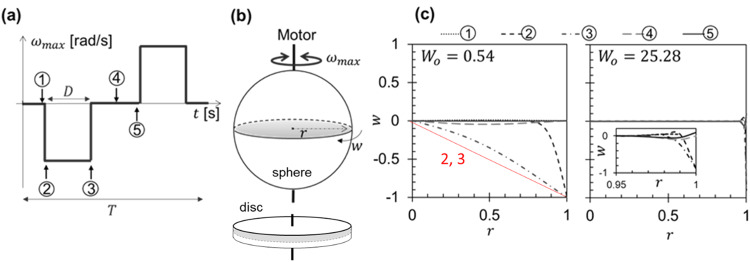
Differences in velocity profiles between a 3D model eye—a sphere—and the ‘eye on a chip’ geometry of Chan et al. [29]. The saccadic motion is modelled as a square wave shown in (**a**), with rotational velocity cycling between $\pm {\omega }_{\max }$, duration *D* and latency time 3*D* so that *T* = 4*D*. **b** shows the geometries: the equatorial plane is shaded. The velocity profiles in (**c**) show the scaled rotational velocity (local value divided by the maximum velocity reached at the wall) at different radial positions. Black loci show the velocity profiles for 0.5 Pa s SO (Womersley number, *Wo* = 0.54) and aqueous, *Wo* = 25.3, at time points indicated in (**a**), for a saccade displacement of 5.4° and ${\omega }_{\max }$ = 200 °s^−1^. Low *Wo* indicates that viscous effects determine the time response. The red locus shows the velocity profile for solid body rotation, which Mulcahy et al. [30] observed in the ‘eye-on-a-chip’ geometry of [29]. The two geometries exhibit significantly difference histories. Based on [21], reproduced with permission.

The 1-D ‘eye-on-a-chip’ presented by Chan et al. [29], featuring a cavity with height 1 mm and diameter 25 mm, allowed retinal ganglion (RGC-5) cells to be grown on the cylindrical walls, but the narrow aspect ratio meant that the liquids were subject to solid body rotation driven by the base and roof of the cavity rather than wall drag (Fig. 6) [30]. The shear rates generated in the wall film (region 2, Fig. 2) then differ significantly from those which arise in the eyeball.

## Conclusions

The advantages of lower viscosity silicone oils, in terms of ease of use (particularly with small gauge vitrectomy instrumentation) need to be offset by their reported greater speed and degree of emulsification when compared with higher viscosity oils. In addition, clinical observation suggests that aside from the viscosity of silicone oil, the speed and degree of emulsification varies widely between patients. Recent research challenges the historic concept of emulsification being due simply to bulk deformation of the silicone reservoir and indicates local disturbance of the contact line across differing biological or physical areas (natural, pathological or surgically induced e.g. scleral buckling) as a potential contributory factor. Further studies of the interaction of such biological and physical influences on the initiation of emulsification at the silicone oil/saline/tissue contact line should inform future strategies to reduce this complication. This is the subject of ongoing research.

### Supplementary information

Supplementary Material
