# Supplementary material for: Refinements in the use of silicone oil as an intraocular tamponade
Source: Eye (Lond). 2024 Mar 28;38(10):1810–5. doi: 10.1038/s41433-024-03027-y (PMC11226643; doi:10.1038/s41433-024-03027-y)
Supplement: Supplementary file 1 — Supplementary Material [file 41433_2024_3027_MOESM1_ESM.pdf]

Eye Refinements in the use of silicone oil as an intraocular tamponade

Supplementary Material

Figure 1 Physical properties and calculation method

Silicone oil diagrams feature a thin aqueous film on the retinal surface – this is expected from the hydrophilic nature of the retinal surface. The location of the contact angle is calculated by a volume balance. Calculation methodology from Eames, I., Angunawela, R.I., Aylward, G.W. and Azarbadegan, A. (2010) A theoretical model for predicting interfacial relationships of retinal tamponades. *Invest. Ophthalmol. Vis. Sci*., 251(4):2243-2247. [https://doi.org/10.1167/iovs.09-4442] implemented in python using scipy and matplotlib. Figures then prepared using inkscape (collating plots into one image and adding contact angles).

Silicone oil interfacial tension taken from Romano, M.R., Angi, M., Romano, V., Parmeggiani, F., Campa, C., Valldeperas, X. and Costagliola, C. (2010) Intraocular pressure changes following the use of silicone oil or Densiron 68 as endotamponade in pars plana vitrectomy. *Clin Ophthalmol*. 26;4:1391-6. [doi: 10.2147/OPTH.S14252] and Erratum in: *Clin Ophthalmol*. 17;4:1521. PMID: 21179224; PMCID: PMC2999554.

Silicone oil densities taken from supplier datasheets

Silicone oil contact angle estimated at 20° from Wang (PhD thesis, 2022)

Sulphur hexafluoride density from PubChem [Internet]. Bethesda (MD): National Library of Medicine (US), National Center for Biotechnology Information; 2004-. PubChem Compound Summary for CID 17358, Sulfur Hexafluoride; [cited 2023 Dec. 24]. Available from: https://pubchem.ncbi.nlm.nih.gov/compound/Sulfur-Hexafluoride

Sulphur hexafluoride contact angle from Rossi, T., Badas, M.G., Angius, F. and Querzoli, G. (2023) Do daily activities affect gas tamponade–retina contact after pars plana vitrectomy?: A computational fluid dynamics study, *Retina*, 43(6): 955-963 [doi: 10.1097/IAE.0000000000003750]

Summary of an investigation of silicone oil tamponade emulsification by contact line motion over pinning features

R. Wang and D.I. Wilson

Department of Chemical Engineering and Biotechnology, University of Cambridge, Philippa Fawcett Drive, Cambridge, CB3 0AS, UK

Key findings

Emulsification is occasionally reported when silicone oil tamponades are employed following retinal repair surgery. The hypothesis that droplets of silicone oil (SO) are generated by the motion of the silicone oil/aqueous interface across oil-wetting features is investigated here using synthetic analogue surfaces with oils of viscosity ranging from 0.01 to 5 Pa s, saline solutions with or without surfactant. Line and dot features with sub-millimetre widths were printed in silane on glass and polymethylmethacrylate (PMMA) surfaces and the contact line moved across it to generate isolated, pinned strands or droplets. The adhesion of these droplets was stronger than the shear forces generated by simulated saccadic motion, except for certain line orientations where breakup of a receding filament generated mobile drops in the bulk aqueous phase. These initial scoping results offer some insight into why emulsification is not found to depend systematically on SO properties.

Background

Emulsification of silicone oil tamponades is a complication of surgery to repair retinal detachment but whose origins remain uncertain and are the subject of much debate. In vitrectomy surgery the vitreous humour – a viscoelastic aqueous collagen gel – is removed to allow access and reattachment of the retina and is replaced after the surgery by a liquid tamponade, which maintains closure of retinal break(s) whilst the tissues heal.

The motion of a three phase (solid-liquid-fluid) contact line (TPCL) and the stability of the thin film formed by an advancing and a receding front has been investigated at length (*e.g*. Keeler *et al*., 2022). For smooth solid surfaces there is often a limiting velocity, U_c, of the contact line above which a thin liquid film will be entrained on a partially wetting surface. Several models exist for the corresponding limiting capillary number, $Ca_c \equiv \mu_L U_c/\gamma$, where μ_L is the viscosity of the liquid (*e.g*. Cox, 1986; see the review by Snoeijer and Andreotti, 2013): the fluid considered is often air and its inertia is not considered. The motion of a contact line between two liquids with similar density but very different viscosities (here, $\nu_{SO}/\nu_{aqueous}$ ~ $O(10^3)$) has received less attention and such studies often consider sessile drops rather than a film. The application of liquid-air models to the emulsification problem is

limited as the velocity and stress fields will be influenced by the fixed volume of each phase. Moreover, Wang *et al*. (2022) did not observe any emulsification at the contact line in their experiments, which featured smooth surfaces.

One of the factors supporting an emulsion mechanism linked to contact line motion is that the retinal surface is heterogeneous, featuring fibril-like protrusions as well as scar tissue and other roughness elements which could potentially act as pinning sites. The latter will differ between patients and could explain why emulsification is observed irregularly, whereas an interface instability mechanism would be expected to be observed more predictably as its occurrence would be determined mostly by chemical factors (and level of fill). Van Gestel *et al*. (2020) summarised recent work on the motion of liquid-air menisci generated by partially wetting liquids moving across topographical surface features. They presented experimental and numerical results for a water-glycerol mixture (μ_L= 0.22 Pa s, 1250 kg m^{-3}, γ = 61 mN m^{-1}, with advancing (receding) contact angles on the substrate and features of 100±2° (71.2±2°) and 88±2° (12.2±2°), respectively. The liquid preferentially wetted the features (circular, square and triangular, with dimensions < L_c and different spacings) and as the contact line withdrew across it (see Figure S1) some liquid remained attached to the feature and give rise to a connecting strand (labelled a meniscus) to the reservoir. Satellite droplets or pearling instabilities could be formed by capillary breakup of the meniscus.

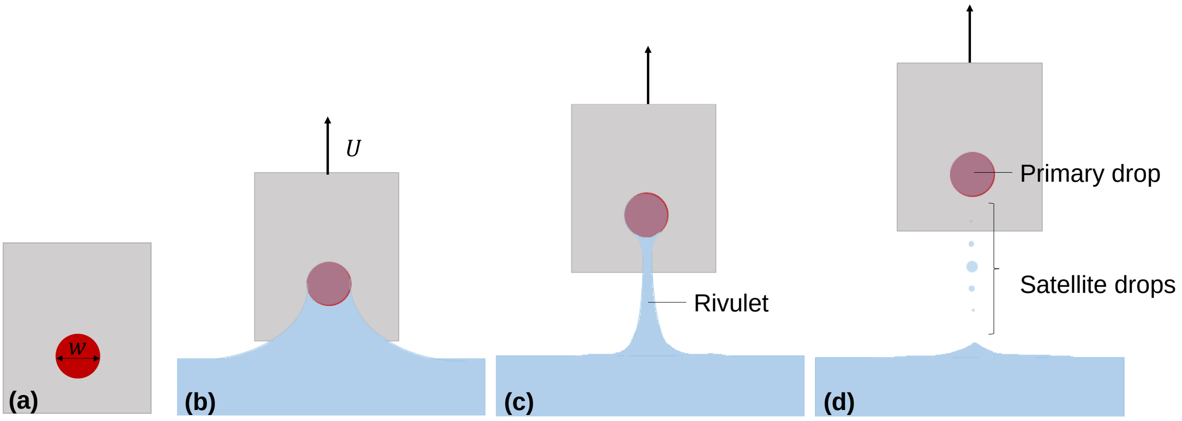

Figure S1. Schematic of rivulet formation as a circular defect (in red, of diameter *w*) is drawn through the interface between two fluids (denser fluid in blue) at velocity *U*.

Rupture of a SO film at a surface feature or pinning point could give rise to isolated droplets, which could subsequently be detached by a shear flow in the aqueous film. This latter scenario, for smooth and even surfaces, has been modelled by Spelt and co-workers (2006, 2008) and experimentally by Seeveratnam *et al.* (2010) as well as Madani and Amirfazli (2014)). Seeveratnam *et al*. studied the

motion of SO droplets on glass (static contact angles, defined in oil, of 140° [advancing]-90°[receding]) with viscosity ratios of order 10-9000, and identified three regimes: no sliding, sliding without detachment, and sliding/crawling with detachment. High values of the viscosity ratio (as encountered with tamponades) resulted in complex deformation, and droplet breakup to give daughter droplets. Madani and Amirfazli considered SOs on PMMA and Teflon surfaces, giving rise to different contact angles (θ~ 90-97° and 66-67°, respectively). The regimes occupied different regions on a plot of capillary number, $Ca_{\mathrm{D}} \equiv \mu_{\mathrm{aq}}U/\gamma$ and a droplet shear group, $G \equiv Ca^2/Re = \mu_{\mathrm{aq}}{}^3U/\gamma^2\rho a$. In this formulation *U* is the mean velocity in their channel flow tests, *a* the initial, undisturbed drop radius. Lu *et al.* (2019) studied the effect of surface wettability on the deformation of sessile kerosene and SO drops subjected to shear flows of water in a similar experimental setup to Seeveratnman *et al.*, for viscosity ratios of 1-400 and θvalues (defined in oil) of 45-118°. They reported that the critical shear rate required to deform a droplet depended more on the wettability and size of the droplet, and less on its viscosity. Relating these studies to tamponades is complicated by the unknown thickness of the aqueous layer present in the eye.

Generation of droplets generated by the passage of a liquid-liquid interface over surface features has not been investigated for the conditions relevant to SO emulsification. This is investigated experimentally here, employing a configuration similar to that employed by van Gestel *et al.*, for a simple set of surface features, *i.e.* dots and lines, on flat substrates.

The characteristic parameters of the eye geometry are as follows: the maximum value of the wall velocity, *U*, initiating contact line motion, is initially of order $R\omega$~ 0.19 m s^{-1} for ω = 5π rad s^{-1} (900 °s^{-1}). This gives a translational capillary number, $Ca_{\mathrm{L}} \equiv \mu_{\mathrm{SO}}U/\gamma$, up to 5 (in the absence of surfactant; up to 25 with surfactant). A second characteristic velocity is that at which the contact line restores to its equilibrium position (*e.g.* following a displacement in the vertical plane: labelled U_{g}, this is measured experimentally here for vertical substrates. In the eye, this velocity will vary locally with position on the retina and the level of fill. The contact angle is determined by the nature of all three phases: Rubowitz *et al.* (2020) reported contact angles for SOs in water on animal retinal surfaces of 19.2°.

In this work, glass and polymethylmethacrylate (PMMA) surfaces were used to investigate the stability of the saline/SO contact line on (*i*) moving flat surfaces and (*ii*) surfaces with local features – dots or lines – of different surface energy, fabricated by contact printing of a silane coating. It was not possible to generate these features within the spherical test devices employed by Wang *et al.* Tests were therefore mostly conducted using flat, rectangular test pieces, which were moved upwards and downwards through an otherwise static SO/aqueous interface. The maximum velocity achievable in

this configuration was 0.04 m s^{-1}, so the full parameter space could not be investigated. The findings reported here nevertheless provide insights into likely mechanisms and are presented to encourage further investigation.

Experimental

Four chemical grade silicone oils were considered, with dynamic viscosities of (approximately) 0.01, 0.5, 1 and 5 Pa s at 20°C. A pH 7.4 saline solution was prepared using deionised water and laboratory grade salts: 30 mM $NaCH_3CO_2{\cdot}3H_2O$ (Alfa Aesar), 3 mM $CaCl_2$, 1 mM MgCl, 111 mM NaCl (all Fisher Scientific), 15 mM $HOC(COONa)(CH_2COONa)_2{\cdot}2H_2O$ and 10 mM KCl (both Sigma Aldrich). 1 wt% Triton TX-100 (Fisher BioReagents, a non-ionic chemical surfactant) was added to the saline to reduce its surface tension, mimicking the presence of surface-active proteins in the eye. The properties of the liquids used are reported in Table S1.

Table S1. Physical properties of liquids at room temperature (20°C).

Liquid		Density ρ (± 1 kg/m^3)	Dynamic viscosity μ (± 0.02 Pa s)	Kinematic viscosity ν (mm^2/s)	Surface tension γ_{LV} (± 2 mN/m)
Saline		1007	0.0017 (± 0.0002)	1.7	73
1 wt% TX-100 in saline		1007	0.0022 (± 0.0002)	2.2	31
Silicone oils	A	973	0.0096	9.9	18
	B	974	0.51	524	18
	C	973	0.92	946	19
	D	976	4.66	4770	19

Rectangular, flat substrates were clamped to the arm of a TA.XTplusC Texture Analyser and moved vertically upwards and/or downwards through a static SO/aqueous interface as shown in Figure S2. The liquid layers were contained in an open cubic PMMA box, one wall of which was inclined at the SO-aqueous-wall meniscus angle so that the SO-aqueous interface was horizontal and a Basler digital camera with a long focus lens could then image the meniscus and the TPCL motion on the substrate.

The substrates were either borosilicate glass microscope slides (Fisher Scientific, UK) or cast PMMA slides (Engineering & Design Plastics, Cambridge, UK) with dimensions 75×25×1 mm. Glass slides were

used as received and not reused. PMMA slides were plasma cleaned in air. The advancing (static) water contact angles in air on the glass and plasma surfaces were 4.1(±3)° and 38(±3)°, respectively.

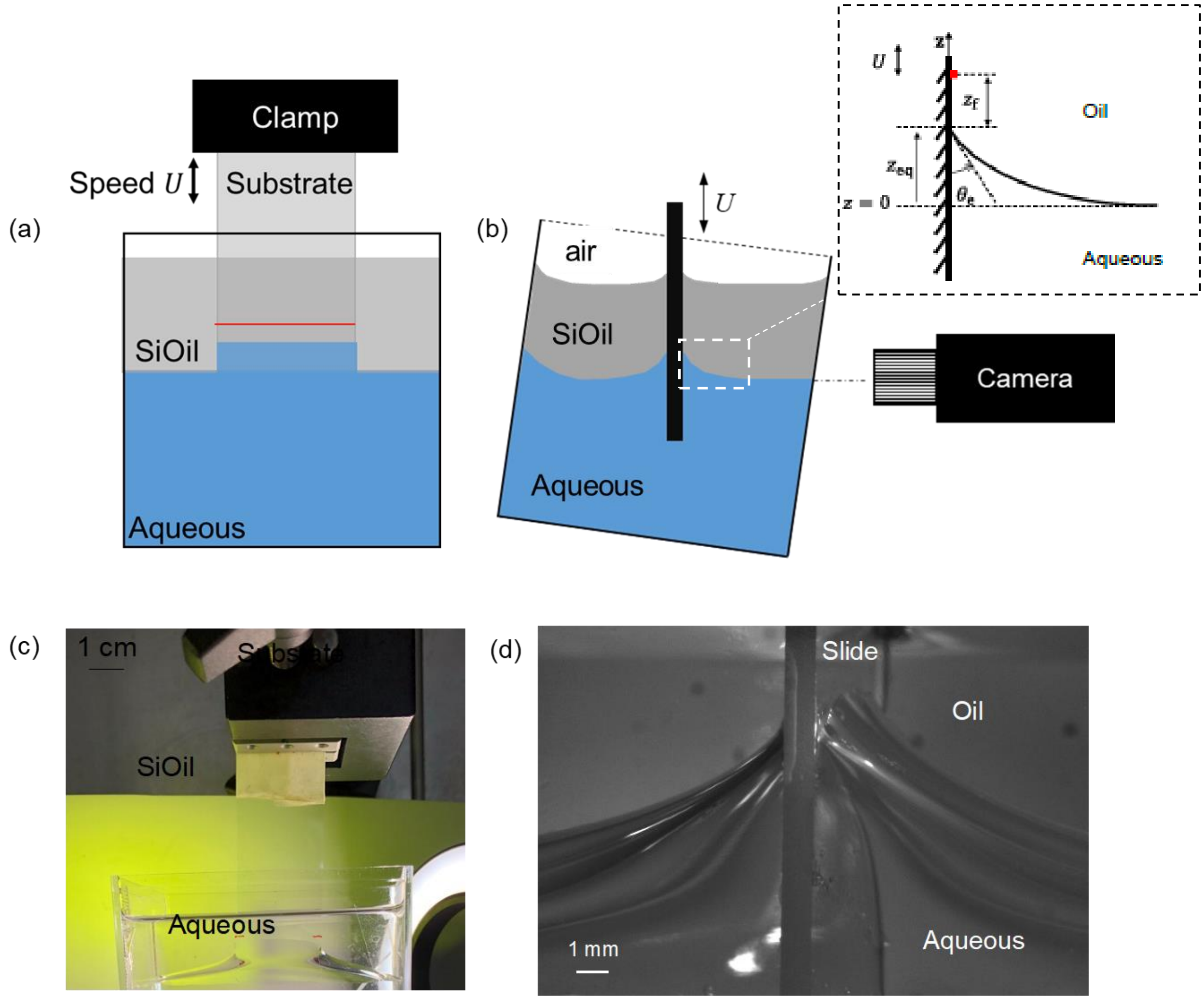

Figure S2 Apparatus for moving contact line studies. (a) schematic, front view; (b) schematic, side view, inset showing meniscus geometry; (c) photograph, front view; (d) photograph, side view.

The surface energy of the substrates was determined from contact angle measurements on small sessile drops (Bond number « 1) with six liquids (dodecane, toluene, dimethyl sulfoxide, ethylene glycol, glycerol and water). Plotting these in terms of the Owens-Wendt theory gave the surface energy values in Table S2. The surface energy of the glass was larger than the plasma-treated acrylic: silane coating the glass gave it a surface energy close to that of dodecane (γ_{LV} = 24.9 mN m^{-1}) and the silicone oils (γ_{LV},= 18-19 mN m^{-1}) rendering it more wetting towards SOs.

Table S2. Surface energy components (in mN m^{-1}) for surfaces and measured advancing contact angles (in degrees) for O – SO (1 Pa s)/air; W – water/air; L – saline solution/air; T – surfactant solution/air. Values in square brackets are those reported by 1 – Fawcett et al. (2004); 2 – Mader-Arndt *et al*. (2014), 3 – Yu *et al*. (2015); 4 – Sharma *et al*. (2019).

Substrate	γ_{SV}^{p} (± 5)	γ_{SV}^{d} (± 3)	γ_{SV} (± 6)	θ_{O}	θ_{W}	θ_{L}	θ_{T}	θ_{WO}	θ_{LO}	θ_{TO}
Glass	42 [35[2]]	21 [29[2]]	63 [64[2]]	9.3 [7[4]]	4.1 [5[4]]	4	5	48 [51[4]]	51	17
Silane coated glass	8 [1[2]]	17 [21[2]]	25 [22[2]]	69	105 [107[2]]	109	52	141	145	70
Acrylic	20 [16[3]]	12 [18[3]]	41 [34[3]]	12	68 [75[3]]	71	22	119	117	69
Plasma acrylic	37 [50[3]]	15 [13[3]]	52 [63]	12	38 [48[3]]	40	8.7	71	77	8.2 [16.4[1]]

Also reported in Table S2 are the static (advancing) contact angles measured for liquid-air and liquid-liquid sessile drops using a Kruss DSA 100 goniometer. Both oil and water had a high affinity for glass in air, with $\theta < 10°$. The water-SO contact angle on glass, of approximately 50°, indicated a stronger affinity for water which was enhanced by adding surfactant to the aqueous phase, giving a value close to that reported by Fawcett *et al*. (1994) for water/SO on retinal surfaces. Silane coating the glass resulted in a hydrophobic surface, which was preferentially wetted by the SO. The contact angle for water in oil was larger than that for PMMA but adding the surfactant made the PMMA more strongly wetting to the aqueous phase.

The meniscus rise on the smooth substrates ranged from 2 to 6 mm and agreed with expected values ($z_{eq} = L_c\sqrt{2(1-\sin\theta)}$). The critical speed for SiOil film entrainment, U_c, was determined by comparing the observed TPCL motion with the known displacement of the substrate when the latter was moved downwards at constant speed. The values obtained were in good agreement with the Cox (1986) model: for example, U_c for entraining a 0.92 Pa s oil film on glass (θ= 0.84) was 6 mm s^{-1} whereas on silane-coated glass (θ = 2.53, more hydrophobic) this decreased to 0.083 mm s^{-1}.

The restoration velocity, U_g, was measured by moving a smooth substrate 5 or 10 mm downwards from its equilibrium position at a velocity of 1, 5, 10 or 40 mm s^{-1} then monitoring the velocity at which the TPCL returned to its initial position.

Printing features

Scanning electron microscopy images obtained by Takano *et al*. (2006) showed topographical features on the retina with estimated widths of 10 μm and lengths ranging from 10- 100 μm. Individual lines and series of dots with the dimensions in Table S3 were selected to reflect these retinal topographical features. Features were printed using polydimethylsiloxane stamps. Silane solution (1 %v/v trichloro(1H,1H,2H,2H-perfluoro-octyl)silane was spread on the stamp and left to dry for at least 5 min. The stamp was then brought into contact with the substrate for 10 s, and the inked pattern left to dry for at least 10 min before use. When loading a stamped substrate, the pattern region was initially held in the upper (SO phase), above the TPCL, while the static meniscus was established. The substrate was then lowered a set distance at velocity U then held at the final position, so that the features either (*i*) passed through the contact line (when $U < U_c$) on the downward step or (*ii*) when $U > U_c$, the contact line passed over the features in the restoration step. In experiments with silane-stamped PPMA and aqueous TX-100 solution, the printed regions detached from the substrate, indicating that chemical bonding would be required to study this combination of surface energies and wettabilities. As a result, only results for silane-stamped PPMA and saline solutions are presented.

Table S3: Dimensions of line and dot features

Labels	Length /mm	Width /mm	Depth /mm	Dot-Dot distance /mm
Line A	30	0.018	0.013	-
Line B	30	0.03	0.026	-
Line C	30	0.05	0.053	-
Line D	30	0.1	0.1	-
Dot 3	-	0.027	0.027	0.5
Dot 4	-	0.027	0.027	1
Dot 5	-	0.05	0.054	0.5
Dot 6	-	0.05	0.054	1
Dot 7	-	0.1	0.1	0.5
Dot 8	-	0.1	0.1	1

Droplets in shearing flow

Two methods were employed to study detachment of droplets formed on the silane printed features. In the first, droplets were formed on the substrate using the dipping action described above and the substrate lowered well into the chamber. Once the contact line had reached its equilibrium state, the substrate was moved up and down at 40 mm s^{-1}, over 10 mm, such that the features did not leave the aqueous phase, exposing the drops to the maximum shear rate that the device could generate.

In the second method, the silane features were stamped on the external wall of a borosilicate glass test tube (radius 6.5 mm) which could be rotated by the stepper motor employed by Wang *et al.* (2022). The tube was filled with mineral oil with similar refractive index to the glass tube to eliminate refraction effects. The tube was lowered slowly into a stratified bath of oil and aqueous solution, promoting oil droplet formation on the features, with the droplets immersed in the aqueous phase. The tube was then subjected to saccadic motion, through an angle of 5-30°, at 3.5-14 rad s^{-1} and a rest period of 0.2 s. The maximum linear velocity, of 91 mm s^{-1} was twice than achievable with the dipping device. Droplets were imaged using the Basler camera at 50 fps.

Results and Discussion

Restoration velocity

Figure S3 shows that the speed at which the contact line returned to its equilibrium position was almost linearly proportional to the vertical displacement of the contact line from the equilibrium position, z_{eq}, for all four SOs tested. Similar behaviour was observed for the other combinations investigated (saline solution on acrylic; TX-100 solution on glass and acrylic, data not reported). This relationship is consistent with U_g being determined by a balance between viscous dissipation at the contact line and hydrostatic driving force, *viz*.

$$C_1 \mu_{\mathrm{SiOil}} U_{\mathrm{g}} \sim C_2 \Delta\rho g \left(z_{\mathrm{eq}} - z\right) \qquad [1]$$

where C_1 and C_2 are geometrical factors which are expected to vary with L_c and θ, while $\Delta\rho g$ did not change significantly between the oils considered here. Figure S4 indicates that $U_{\mathrm{g}} \approx m\left(z_{\mathrm{eq}} - z\right)$: rewriting [1] as

$$\mu_{\mathrm{SiOil}} m = \frac{C_2 \Delta\rho g}{C_1} \qquad [2]$$

allows the factors determining C_1 and C_2 to be investigated. Figure 6 shows that for each solid surface, $\frac{C_2 \Delta\rho g}{C_1} \sim \left(\frac{\mu_{\mathrm{SO}}}{\gamma_{\mathrm{SO/aq}}}\right)^{1/2}$, indicating that both viscous dissipation and capillary forces both played a role. Further analysis of this behaviour was not undertaken.

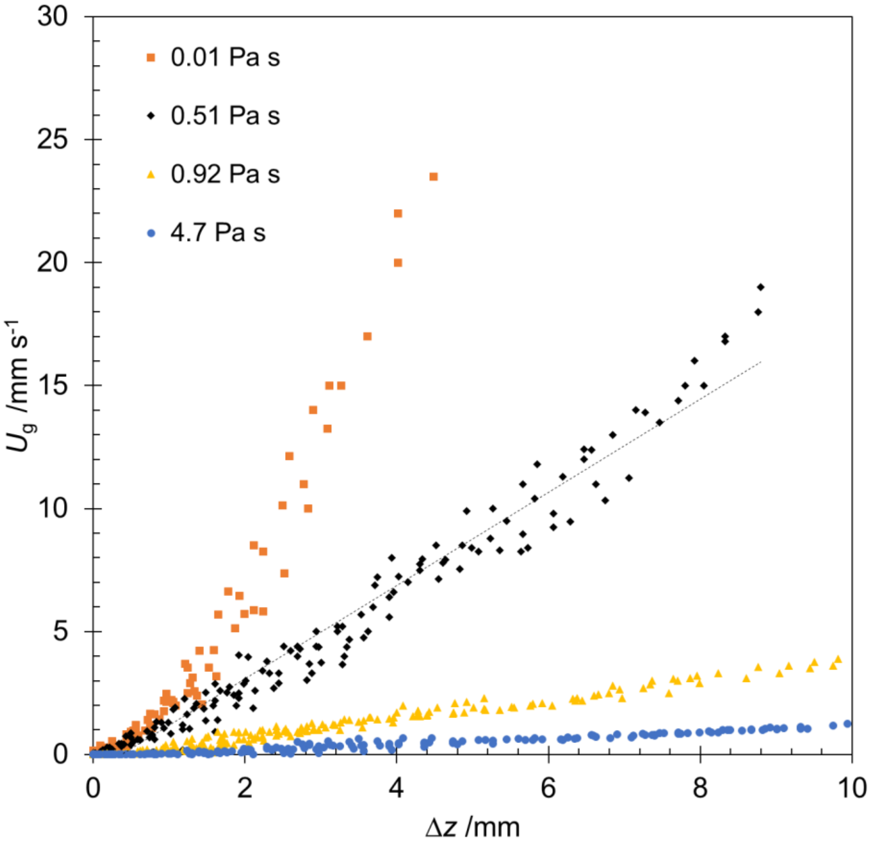

Figure S3. Effect of displacement from equilibrium position, $\Delta z = (z_{\mathrm{eq}} - z)$ on restoration velocity for different SOs. Conditions: glass, saline: initial descent 10 mm at 10 mm s^{-1}. Construction line shows fit to the data to obtain gradient *m*.

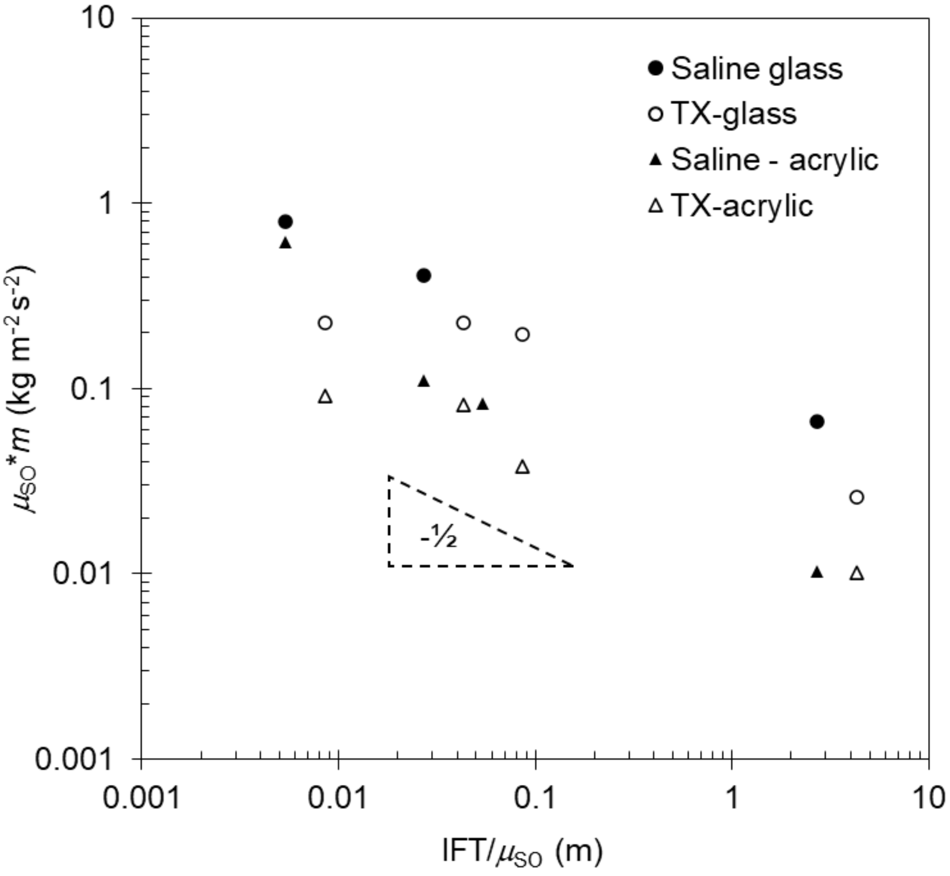

Figure S4 Effect of ratio of interfacial tension to SO viscosity on the ratio of viscous dissipation at the TPCL to hydrostatic pressure during the restoration stage on vertical substrates. Circles – glass; triangles - PMMA. Open symbols, SO-(1 wt.% TX-100 in saline); solid symbols, SO-saline.

Circles

Figure S5 shows an example of the pinning of the contact line on a horizontal row of circular features (Dot 8) on glass (the silane coating did not attach reliably to the PMMA substrates). In this case the substrate was moved down quickly (U > U_c) and the contact line passed over the features in the restoring stage. Rivulets are evident in Figure S5(*b*): these eventually ruptured, leaving isolated droplets on the stamped region, as reported by van Gestel *et al*. The diameter of the droplet, D_{oil}, was measured and is compared with the value expected for a sessile drop of diameter w, $D_{\text{oil}} = w\big(1 + tan(\theta_{Oi} - \pi/2)\big)$, in Figure S6. There is good agreement with this geometric result. None of the drops were dislodged by subsequent shearing, indicating that attachment of the SO to the silane was stronger than the viscous forces that the experimental apparatus was able to generate. Tests with other Dots gave similar results.

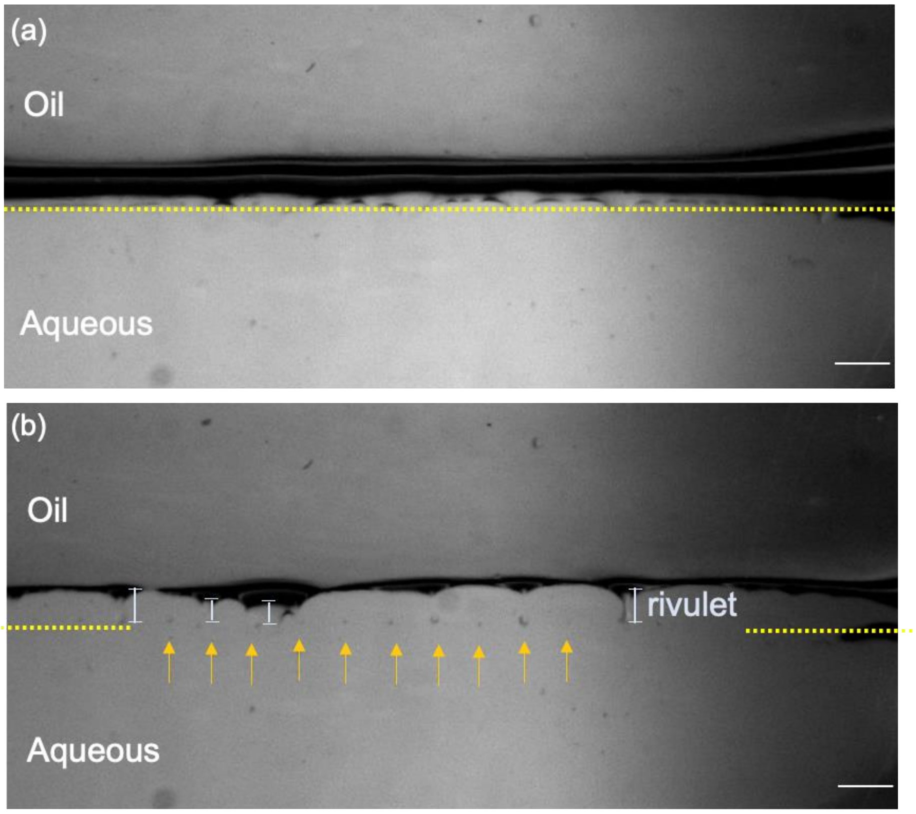

Figure S5 Photographs showing (a) pinning of the TPCL (TX-100 0.92 Pa s SO) over horizontal line of dot features printed on glass (Dot 8, indicated by the yellow dotted line); (b) formation of rivulets, then droplets at pinning sites (droplets indicated by arrows). Substrate motion: single dip and restoration, U = 5 mm s^{-1}s. Scale bar = 1 mm.

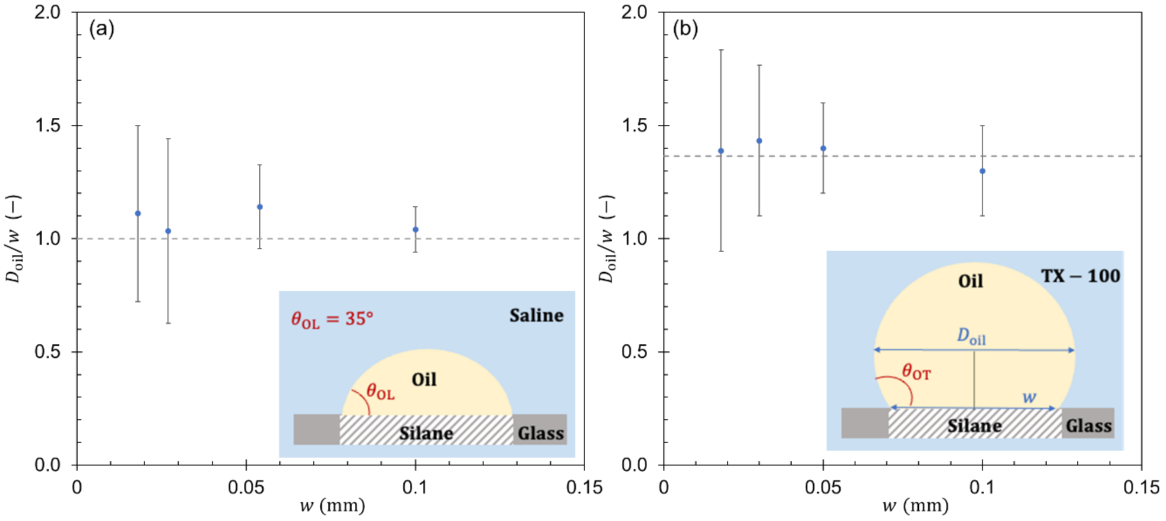

Figure S6 Effect of dot width on entrained oil droplet diameter, glass substrates. 092 Pa s SO and (*a*) saline; (*b*) 1 wt.% TX-100 in saline. Error bars indicate standard error. Insets shows shape of sessile drop generated by attachment at the silane/glass boundary. Horizontal dashed line shows geometric result, $D_{\text{oil}} = w\big(1 + tan(\theta_{Oi} - \pi/2)\big)$.

Vertical lines

Figure S7 shows the behaviour observed with silane lines on glass when the initial downward velocity, U, was (*b*) less than, or (*c*) greater than, U_c. At slow speeds the feature moved through the TPCL and any SO attached to it dewetted periodically, generating a series of short strands along its length. At higher speeds, the TPCL moved over the line feature during the restoration stage, causing local dewetting and again generating a series of short SO strands. The strands were characterised by their length, l_s, and the spacing between strands, l_{ss}. Figure S8 shows that the strand length was determined by the substrate dipping speed, whereas the spacing between strands was not. The speed here is expressed in terms of Ca_L, evaluated using the SiOil viscosity. At higher speeds, inertia favoured the formation of longer strands while at slow speeds capillary forces encouraged the formation of spherical caps ($l_s \sim w$). When the saline contained the surfactant, the strands formed truncated spherical drops on the line feature (Figure S7(*f*)). This is consistent with the measured increase in the contact angle (defined in SO on silane) from 35° to 110°, promoting dewetting of the oil. In this case $l_s \sim w$, independent of Ca_L, and l_{ss} decreased weakly, from 5 to 4, over the range $10^{-4} \leq Ca_{\mathrm{L}} \leq 10^{-2}$ (data not reported).

Similar trends were observed with saline-SO-PPMA (no surfactant). The substrate's lower surface energy (and subsequently larger contact angle, Table II) resulted in a lower U_c value, of 1.5 mm s^{-1}. At slow speeds, e.g. U = 0.01 m/s (Ca = 4.4×10^{-7}), the SO did not form strands. At other speeds, strands with $l_s/w \sim 50$ and spacing $l_{ss}/w \sim 0.2$ (similar to glass) were formed as the TPCL passed over the silane feature.

Neither substrate/strand combination gave droplets that detached during extended shearing.

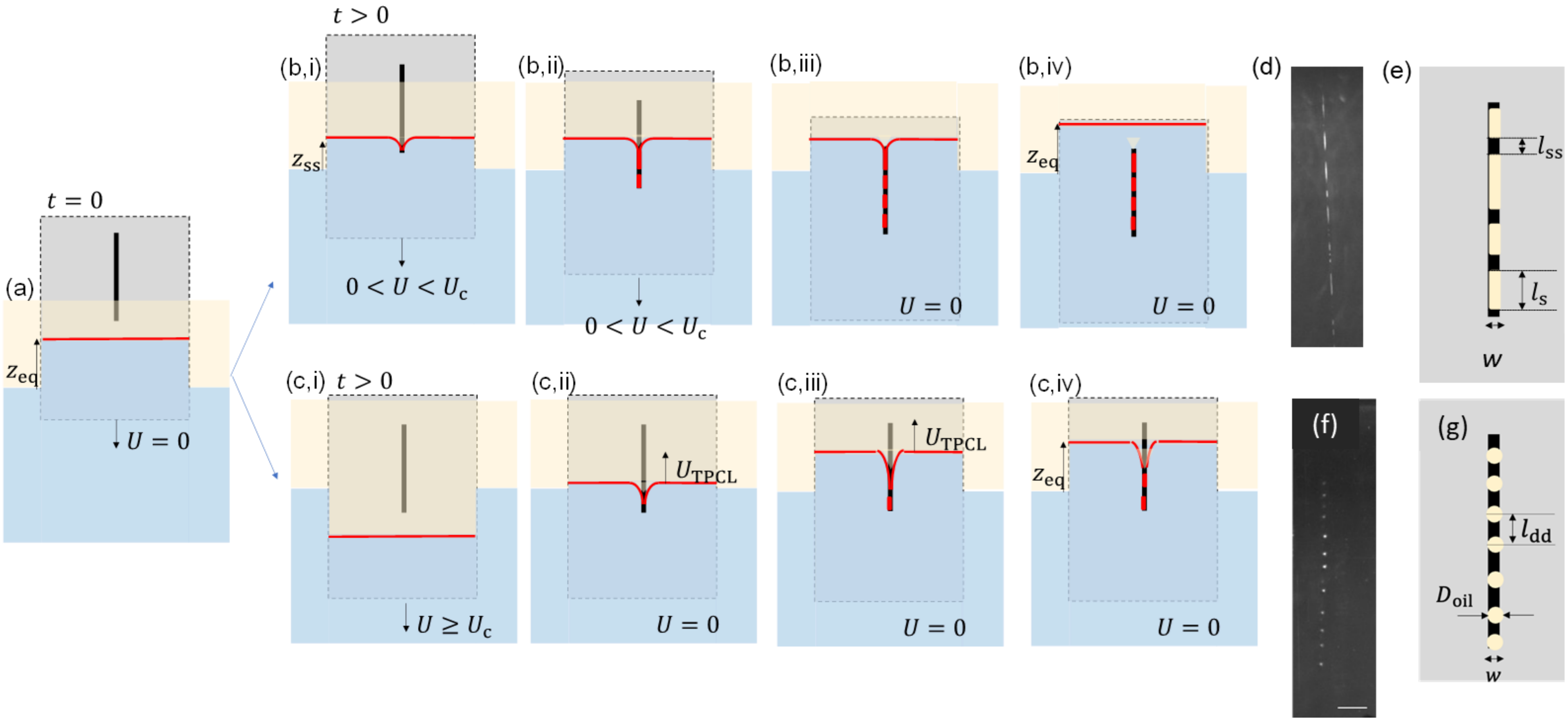

Figure S7: Schematic representations of TPCL motion across silane features (black vertical line) printed on the substrate (grey rectangle). Cream – SO, blue – aqueous solution, red line – TPCL, red segments – SO strands. (*a*) Initial state: z_{eq} is the meniscus height rise; (*b*) the slide moves downwards slowly: the TPCL moves to height, $z_{ss} < z_{eq}$, and SO strands are formed on the line feature; (*c*) when $U > U_c$ the TPCL moves over the line during the restoration stage; (*d*) example of 0.92 Pa s SO strands generated with silane; (*e*) strand dimensions; (*f*, *g*) photograph and dimensions of 0.92 Pa s SO drops formed with 1 wt% TX-100 solution.

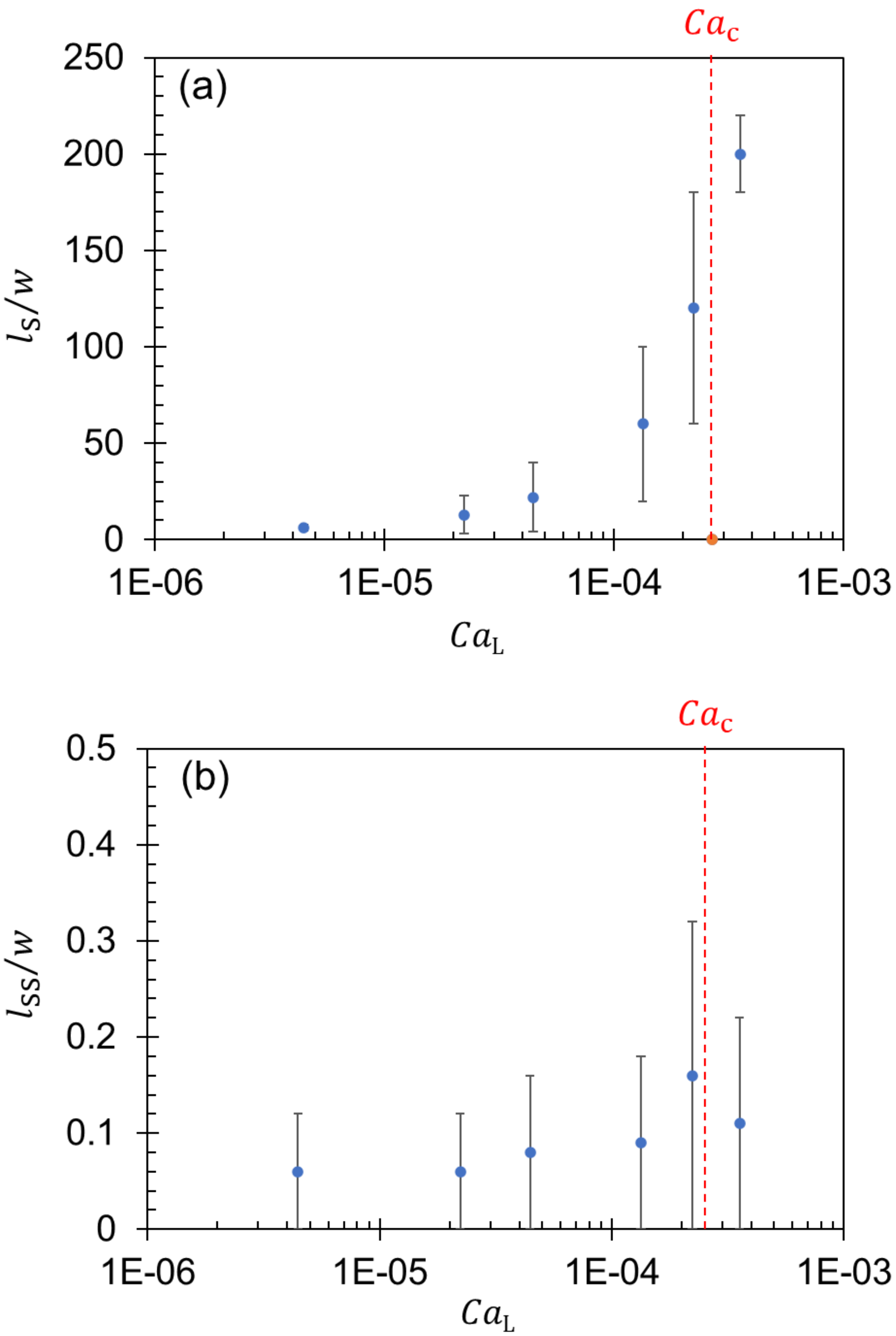

Figure S8. Effect of substrate speed, expressed as Ca_L, on entrained oil segment dimensions generated on vertical silane line features (Figure S7) - (*a*) length, and (*b*) spacing. Case: line C, 0.92 Pa s SO/saline. Vertical red dashed line indicates $Ca_c(\text{oil}) = (2.7 \pm 0.1) \times 10^{-4}$, the condition for liquid entrainement for $\theta = 0.83$ rad. Above Ca_c strands were generated during the resoration stage.

Horizontal lines

Figure S9 summarises the behaviour observed with horizontal line features. At higher immersion speeds, the TPCL was pinned to the descending line feature (Figure S9(*c*)), whereas at lower speeds (Figure S9 (*b*)) the restoring TPCL stopped at the line feature. The meniscus then started to detach from the ends of the line, generating an oil thread on the feature as the meniscus narrowed. The thread subsequently broke into smaller strands. The length and spacing of the pinned oil strands followed similar trends and values as the vertical line features, on both substrates.

Before the TPCL completely detached from the line feature, the meniscus formed a liquid bridge between the line feature and the bulk interface. This rivulet eventually broke up into a primary drop on the feature and satellite droplets on the substrate vertically above the feature, between the line and the restored meniscus in a manner reminiscent of break-up in a filament stretching rheometer used to determine extensional viscosity. This result is consistent with Van Gestel *et al*.'s observations of liquid rivulet behaviour on patterned surfaces, with rivulets detaching from the edges of the pattern and forming primary and satellite droplets in the wake.

In all tests with the 0.92 Pa s SO, the primary and satellite droplets remained pinned to the silane and substrate surfaces, respectively and did not detach over time, despite shearing. The less viscous tamponade oil (0.51 Pa s) gave a more mobile meniscus. Figure S10 shows the receding film splitting at several points on the line feature, rather than progressing inwards from the ends, forming multiple rivulets, then primary drops on the line and secondary drops on the substrate after the filament ruptured. In this case the satellite drops were observed to subsequently rise in the aqueous phase as a combined result of buoyancy and shear from by the wake of the receding meniscus. This was observed for all line widths for this motion ($U > U_c$, 10 mm displacement, z_f = 1 mm). A similar sensitivity to SO viscosity was reported by Seeveratnam *et al*.: the conditions for detachment are determined by the shear generated on the droplet, its deformability and the strength of adhesion to the substrate.

These observations indicate that formation of mobile secondary droplets requires the presence of a region that the SO wets preferentially, oriented such that a meniscus will form as the TPCL passes over it, the dynamics of which result in a filament that will rupture to release secondary droplets. The latter component is consistent with the finding of Williams *et al*. (2010), that emulsification was observed less frequently in SOs with high extensional viscosity. This hypothesis requires further investigation, focusing on the difference in wettability of feature and the substrate by silicone oils, for surfaces more representative of the retina than the synthetic analogues employed here.

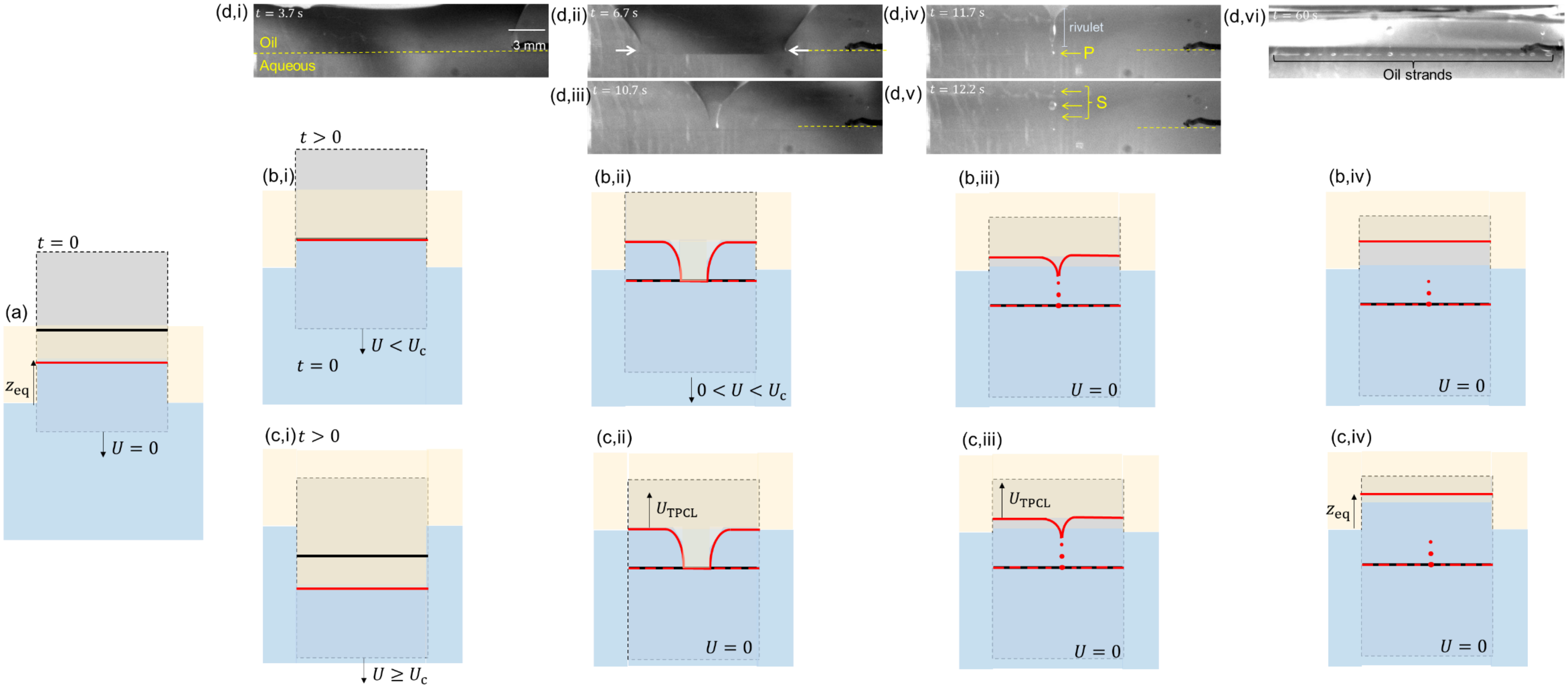

Figure S9 TPCL motion across horizontal line features (black line) printed on the substrate (grey rectangle). Cream – SO layer, blue – aqueous solution, red line – TPCL, red segments – SO strands. (*a*) Initial state; (*b*) the slide moves downwards slowly: the meniscus is pinned to the line, then detaches, forming patches and a meniscus (ii), leading to a filament (iii) which ruptures, creating daughter droplets (iv). (*c*) When $U > U_c$ the TPCL moves over the line during the restoration stage; (*d*) photographs: yellow dashed lines indicate location of line feature. P – primary drop; S – satellites. [0.92 Pa s SO/saline; U = 10 mm s^{-1}, glass, 10 mm initial downward displacement].

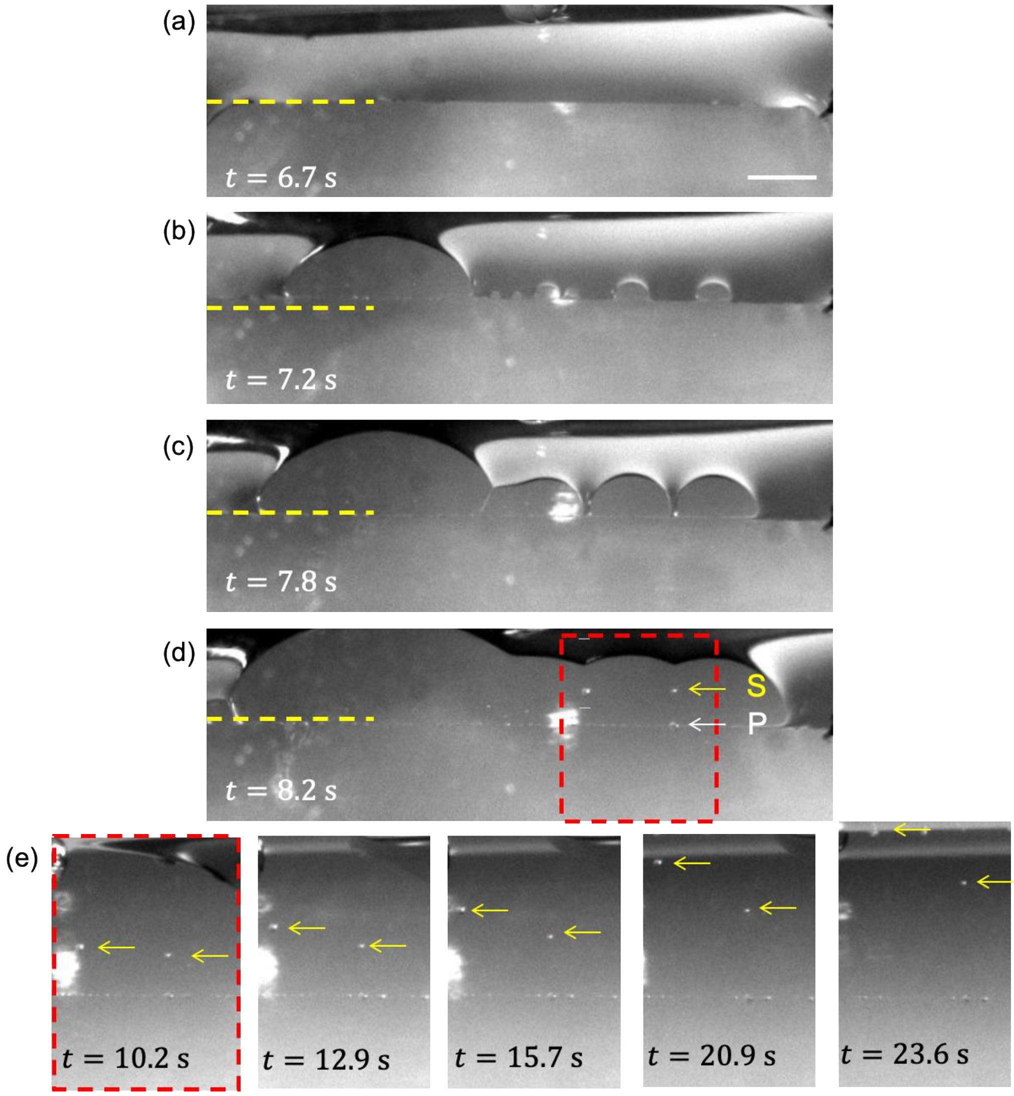

Figure S10 Images of a receding 0.51 Pa s SO/TX-100 TPCL on glass dewetting (restoration stage) from a horizontal Line D feature (location indicated by the yellow dashed line). (*b*) formation of several menisci and (*c*) rivulets. (*d*) Rivulet breaks into primary (P, pinned on line) and satellite (S) oil drops. (*e*) Satellite drops (marked by yellow arrows) detach and rise in the aqueous phase. Scale bar = 2 mm.

Shear driven detachment

The propensity for shear in the aqueous phase to dislodge droplets formed on silane features on vertical rectangular substrates was tested by waiting for the meniscus to reach its equilibrium position (well above the sites) after droplets had been formed by one of the modes described above, then moving the substrate up and down repeatedly through a distance of 5 or 10 mm, at speeds of 20 or 40 mm s^{-1}, with latency periods of 50 or 200 ms. The shear induced by the accelerating wall on the

adjacent aqueous phase was not sufficient to dislodge the droplets from the silane patches in all the tests conducted here. The distance that the shear penetrates the aqueous phase can be estimated from the Stokes boundary layer thickness (Erdogan, 2000), $\delta_{\mathrm{St}} = \sqrt{2\nu/\Omega}$, where $\Omega/2\pi$ is the frequency of the plate oscillation. This gave δ_{St} values of 0.44 mm for the aqueous phase, which is larger than the drop dimension. The shear rate around the drop was then estimated as U/δ_{St}.

Higher surface velocities were investigated using glass test tubes with vertical line features, subjected to saccadic motion for (*i*) 1 Pa s SiOil-saline, and (*ii*) 1 Pa s SiOil-surfactant solution, with angular displacements of 10-50°, $400 \le \omega \le 1000$ °s^{-1} and latency periods of 50 and 200 ms, giving a maximum surface velocity of 0.17 m s^{-1} and estimated shear rate of 360 s^{-1}.

Comparison of the conditions generated in these tests with those employed by Seeveratnam *et al.* (2010) and Lu *et al.* (2019) requires revisiting the former's dimensionless group, *G*,

$$G \equiv \frac{Ca^2}{Re} = \frac{\mu_{aq}^3 \bar{U}}{\gamma^2 \rho_{aq} a} \qquad [3]$$

Both groups employed laminar flows through rectangular duct of height *H*, so the shear rate at the wall can be approximated as $2\bar{U}/H$ and the capillary stress in the droplet is γ/a. The droplet capillary number is then

$Ca_{\mathrm{D}} = 2\mu_{\mathrm{aq}} Ua/\gamma L$, where L is either H or δ_{St}. Assuming local inertia near the drop to be ~ $\rho_{aq}\bar{U}^2$, an alternative form of *G* for these workers' results is

$$G_{\mathrm{c}} \equiv \frac{Ca_{\mathrm{D}}^2}{Re} = \frac{\left(\frac{viscous\ stress}{capillary\ stress}\right)^2}{\left(\frac{inertia}{viscous\ stress}\right)} = \frac{\left(\frac{2\mu_{\mathrm{aq}}\bar{U}}{H}\right)^3}{\left(\frac{\gamma}{a}\right)^2 \left(\rho_{\mathrm{aq}}\bar{U}^2\right)} = 8\left(\frac{a}{H}\right)^3 G \qquad [4]$$

Alternately, the local inertia near the group can be estimated as $\rho_{aq}\bar{U}^2\left(\frac{a}{H}\right)^2$, giving

$$G_{\mathrm{c}}' = 8\left(\frac{a}{H}\right) G \qquad [5]$$

The hydrodynamic conditions for the conditions employed in this work are compared with those studied by Seeveratnam *et al.* (2010) in Table S4. Whereas there is some overlap between the ranges of G_c and G_c' values, the Ca_{D} values differ noticeably. In particular, the dipping device did not access the shear conditions studied by Seeveratnam *et al.*, while the test tube saccades only probed the lower end of their range. When their regime map is plotted in terms of these parameters, *e.g.* Figure S11 using G_c, the test tube and dipping plate conditions lie comfortably in the no-detachment zone. The

lack of droplet detachment reported above is therefore consistent with previous work in this area, albeit for a very different flow mode.

Table S4 Summary of shearing conditions generated during this work and those reported by Seeveratnam *et al*. (2010) using duct flow to generate fluid shear.

	Test tube cycling		Dipping tests		Seeveratnam *et al*. (2010)
	Saline	TX-100	Saline	TX-100	
$Ca_{\mathrm{D}}/10^{-4}$	1.5-15	9.9-99	0.034-0.34	0.23-2.3	1.2-340
$G_{\mathrm{c}}/10^{-11}$	5.6-56,000	0.026-2.6M	0.011-260,000	5.9-59,000	0.13–1,300
$G_{\mathrm{c}}'/10^{-7}$	4.3-430	20-45,000	0.01-25	45-450	0.1–25

This finding suggests that further tests should be conducted, at higher speeds, to see if this would cause the pinned droplets or strands to detach. The validity of the estimated of the shear rate based on the Stokes layer thickness would also need to be verified. The behaviour will also be determined by the difference in surface energies between the substrates, effectively the work of adhesion, and some thought is needed to identify materials that will replicate the conditions on the retinal surface, both in terms of surface energies and morphology.

The above hypothesis assumes that emulsification arises from dislodging the SO drop from a patch of favourably wetting material, and requires the initial formation of stably pinned patches. The observation that SO droplets *can* be generated in the aqueous phase by the relatively slow motion of the TPCL across a surface feature at a 'favourable' orientation, rather than the equivalent of brute force on pinned drops, suggests another pathway which has not been considered previously. The need for a favourable orientation in addition to the physico-mechanical conditions offers an explanation to why SO emulsification cases do not follow consistent patterns.

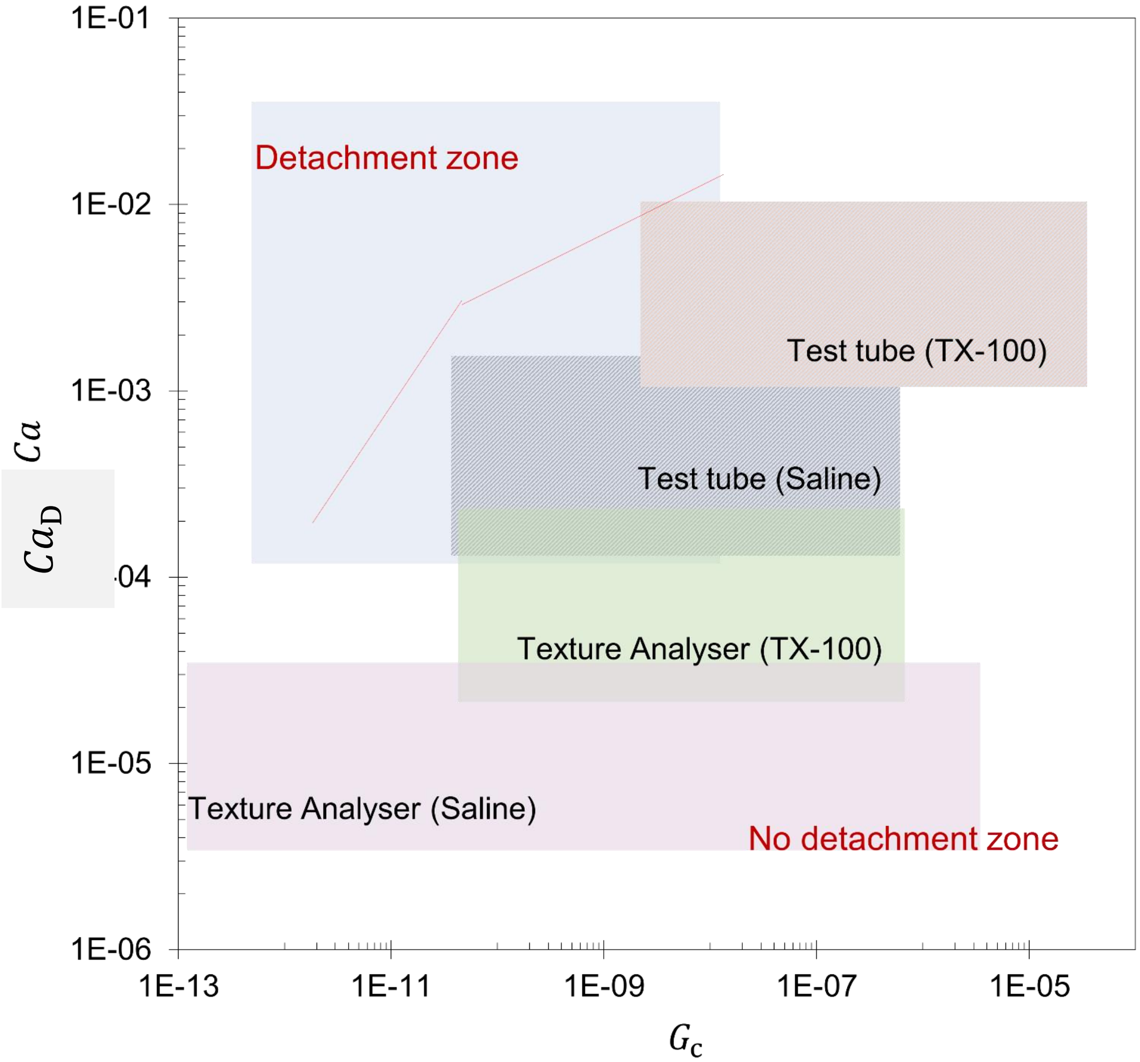

Figure S11: Regime map of oil droplet behaviour with each coloroured rectangle representing the value range of the experimental conditions. Locus shows boundary between detachment and non-detachment reported by Seeveratnam *et al.* (2010) for a 0.92 Pa s SO oil on glass.

Acknowledgements

No external funding was received. A PhD studentship for RW from the WD Armstrong Fund from the University of Cambridge is gratefully acknowledged.

Notation

Roman

a	Droplet radius, m
C_i	Constant, various units
Ca_c	Capillary number, thin film, critical value, -
Ca_L	Capillary number, translation, thin film, -
Ca_D	Capillary number, droplet
D_{oil}	Entrained drop diameter, m
G	Droplet shear group
g	Gravitational acceleration, m s^{-2}
l_s, l_{ss}	Strand length, spacing, m
L_c	Capillary length, m
m	Gradient, Figure 5, s^{-1}
R	Radius, m
Re	Reynolds number, -
T	Saccade duration, s
U	Mean or plate velocity, m s^{-1}
U_c	Critical velocity, m s^{-1}
U_g	Restoring velocity, m s^{-1}
We	Weber number, -
Wo	Womersley number, -
w	Feature width, m
z	Vertical co-ordinate, m
z_{eq}	Equilibrium meniscus position, m

Greek

δ_{St}	Stokes boundary layer thickness, m
γ	Interfacial tension, N m^{-1}
γ^{p}	Interfacial tension, polar component, N m^{-1}
γ^{d}	Interfacial tension, dispersive component, N m^{-1}

μ Dynamic viscosity, Pa s

ν Kinematic viscosity, $m^2 s^{-1}$

ρ Density, kg m^{-3}

θ Contact angle, rad

Ω Oscillation frequency, s^{-1}

ω Angular velocity, rad s^{-1}

Acronyms

PMMA Polymethylmethacrylate

SO Silicone oil

TPCL Three phase contact line

References

Cox, R.G. (1986) The dynamics of the spreading of liquids on a solid surface. Part 1. Viscous flow, J. *Fluid Mech*., 168, 169–194.

Ding, H. and Spelt, P. (2008) Onset of motion of a three-dimensional droplet on a wall in shear flow at moderate Reynolds numbers, *Journal of Fluid Mechanics*. Cambridge University Press, 599, pp. 341–362. doi: 10.1017/S0022112008000190.

Erdogan, M. E. (2000) Note on an unsteady flow of a viscous fluid due to an oscillating plane wall, *Intl. J. Non-Linear Mech*., 35(1), 1–6.

Fawcett, I.M., Williams, R.L. and Wong, D. (1994) Contact angles of substances used for internal tamponade in retinal detachment surgery, *Graefe's Arch. Clin. Exp. Ophthalmol*. 232, 438–44.

Keeler, J.S., Lockerby, D.A., Kumar, S. and Sprittles, J.E. (2022) Stability and bifurcation of dynamic contact lines in two dimensions, *J. Fluid Mech*., 945, A34.

Lu, H., Xu, X., Xie, L., Wang, H., Sun, G., Yang, Q. (2019) Deformation and crawling of oil drop on solid substrates by shearing liquid, *Chem. Eng. Sci*., 195, 720–729.

Madani, S. and Amirfazli, A. (2014) Oil drop shedding from solid substrates by a shearing liquid, *Colloids Surfaces A: Physiochem. Eng. Aspects*, 441, 796-806.

Mader-Arndt, K., Kutelova, Z. Fuchs, R., Meyer, J, Staedler, T., Hintz, W., Tomas, J. (2014) Single particle contact versus particle packing behavior: Model based analysis of chemically modified glass particles, *Granular Matter*, 16(3), pp. 359–375.

Rubowitz, A., Ayalon, A., Roy, P.K., Shoval, S., Legchenkova, I. and Bormashenko, E. (2020) Study of wetting of the animal retinas by water and organic liquids and its implications for ophthalmology, *Coll. Surf. B. Biointerfaces*, 195, 111265.

Seeveratnam, G.K., Ding. H., Michel, O., Heng. J.Y.Y. and Matar, O.M. (2010) Laminar flow deformation of a droplet adhering to a wall in a channel, *Chem. Eng. Sci*., 65, 4523-4534.

Sharma, M., Roy, P.K., Pant, R. and Khare, K. (2019) Sink dynamics of aqueous drops on lubricating fluid coated hydrophilic surfaces, *Coll. Surf. A: Physio. Eng. Aspects*, 562, 377-382.

Snoeijer, J.H. and Andreotti, B. (2013) Moving contact lines: scales, regimes, and dynamical transitions, *Ann. Rev. Fluid Mech*., 45, 269-292.

Spelt, P. (2006) Shear flow past two-dimensional droplets pinned or moving on an adhering channel wall at moderate Reynolds numbers: a numerical study, *Journal of Fluid Mechanics*. Cambridge University Press, 561, pp. 439–463. doi: 10.1017/S0022112006000954.

Takano, A., Hirata, A., Ogasawara, K., Sagara, N., Inomata, Y., Kawaji, T. and Tanihara, H. (2006) Posterior vitreous detachment induced by nattokinase (Subtilisin NAT): A novel enzyme for pharmacologic vitreolysis, *Inv. Ophth. Vis. Sci*., 47(5), 2075–2079.

Van Gestel, M.A.C., He, B. and Darhuber, A.A. (2020) Formation of residual droplets upon dip-coating of chemical and topographical surface patterns on partially wettable substrates, Chem. Eng. Sci., 227, 115832.

Wang, R., Tsai, J.-H., Snead, M., Alexander, P. and Wilson, D.I. (2022) Stability of the interface between two immiscible liquids in a model eye subject to saccadic motion, *J. Biomed. Eng*., 144(5), 051004.

Williams, R.L., Day, M., Garvey, M.J., English, R., and Wong, D. (2010) Increasing the extensional viscosity of silicone oil reduces the tendency for emulsification, *Retina*, 30(2), 300–304.

Yu, H., Chong, Z.Z., Tor, S.B., Liu, E., Loh, N.H. (2015) Low temperature and deformation-free bonding of PMMA microfluidic devices with stable hydrophilicity via oxygen plasma treatment and PVA coating, *RSC Advances*, 5(11), pp. 8377–8388.
